# Sheathed fixation improves BASHTI technique in an anterior cruciate ligament reconstruction

**DOI:** 10.1177/09544119231153198

**Published:** 2023-02-11

**Authors:** Amirhossein Borjali, GholamHossein Farrahi, Mahmoud Chizari

**Affiliations:** 1 Department of Mechanical Engineering, Sharif University of Technology, Tehran, Iran; 2 School of Physics, Engineering and Computer Science, University of Hertfordshire, Hatfield, UK

**Keywords:** ACL reconstruction, BASHTI technique, core bone, sheath, implant-less, fixation strength

## Abstract

Bone and Site Hold Tendon Inside (BASHTI) technique is an implant-less surgical methodology used for anterior cruciate ligament (ACL) reconstruction. It has some clinical advantages, such as speeding up the healing process. Since the force required to insert the core bone inside the tunnel may damage the core bone and affect the fixation process, the study aims to investigate the strength of fixation of BASHTI technique using proposed sheathed core bones. Experimental tests were performed to evaluate the biomechanical strength of the fixation. Synthetic bone combined with bovine tendons as a graft was used. Polymers were used to create the sheath for mechanical testing. The results showed that fixation strength and stiffness in PTFE sheath with 0.1 mm were 343.86 N and 114.62 N/mm and in PVC sheath with similar thickness, 235.95 N, and 93.36 N/mm. Subsequently, 0.2 mm PTFE sheaths were tested in two different sections: incomplete fixation and complete fixation. The strength and stiffness of the first section were 221.6 N and 66.99 N/mm and for the second section 420.02 N and 126.16 N/mm. Using sheath facilitates the fixation process in BASHTI technique. The 0.1 mm PTFE sheath and 0.2 mm PTFE sheath with complete fixation provide higher fixation strength than other groups. The outcome showed that engaged length has a direct effect on the fixation strength. The BASHTI technique offers an implant-less organic ACL reconstruction method that can improve the fixation method and speed up the healing process.

## Introduction

During anterior cruciate ligament (ACL) reconstructions, various methods are used to fix the graft tissue in bone. The most common method is the use of metal and polymer implants, such as Interference screws.^1,2^ However, these methods are associated with an increase in preparation cost, inflammatory response, possible abscesses and germ production,^3–5^ the possibility of corrective surgery and interfering with tibia and femur reconstruction,^6^ divergent screw placement, possible impingement and abrasion,^7^ imaging problems after surgery, and tunnel enlargement.^8^

Press-fit is another technique that uses bone-patellar tendon-bone (BPTB) graft. The bone is shaped and fixed in the femoral and tibial tunnels. In this method, the patellar tendon is used because of the need for bone plugs at the two ends of the graft tissue. Therefore, the length of the graft tissue used for fixation is limited and depends on the distance between two bone plugs on its ends. Also, due to bone-on-bone contact in the fixation area, slipping off the bone plug in the tibial and femoral tunnels is possible. Since the bone plugs are extracted from the patella and tibia, the patient may experience pain at the harvested site. As a matter of fact, the risk of osteoarthritis in patients using BPTP grafting for ACL reconstruction is higher than in patients using hamstring tendon as a graft for surgery.^9^

Bone and Site Hold Tendon Inside (BASHTI) technique is a novel method in which no implants are used.^10^ The BASHTI technique, unlike press-fit methods, uses the core bone taken from the tunnel to fix the tendon graft so that there is no need to take a segment from another side of the body. Indeed, the implant-less functionality of this technique has many benefits, including improving the treatment period, reducing the costs, and inflammatory responses.^10–13^ The fixation procedure in this method is similar to the interference screw. By using the core bone plug, the graft tissue compresses against the tunnel wall and the friction created between the tunnel wall and graft tissue prevents graft tissue from slipping out of the tunnel.^10,11,13,14^ It also uses the hamstring tendon as graft tissue which does not have the aforementioned problems observed in the patellar graft.^9^ In this method, the tunnel site in the tibia and femur is cut by a cannulated drill bit, which can be a custom made drill bit that removes the core bone from the tunnel with a minimum gap.^10,11,13,14^ Following the graft insertion inside the tunnel, the core bone is pushed back to the tunnel to compress the graft against the tunnel wall.^10,11,13,14^

The first study about this technique showed that there is no significant difference between the fixation strength of BASHTI groups and interference groups.^10^ Another study was conducted by Dehestani et al.^15,16^ to investigate the effect of bone density on the mechanical properties and fixation strength of ACL reconstruction. Their results showed that BASHTI technique is more suitable for individuals with high density bones, 240  and 320 kg/m^3^, which is related to the young and middle-aged patients. Furthermore, due to the notable impact of geometrical variations on mechanical properties,^13,17–19^ the geometrical parameters (i.e. tendon and core bone diameters) on the fixation strength of BASHTI technique were investigated. The results showed that the tendon compression and core bone diameter significantly affect the mode of graft failure and fixation strength.

Due to the high friction force between the core bone plug and the tunnel wall, a massive insertion force during fixation is required. Consequently, the insertion force may lead to a fracture on the core bone and could cause improper fixation.^11,13–16^ To tackle this problem, the friction in the fixation process should be reduced. So, using a sheathed core bone is proposed.

The purpose of this study is to examine the effect of sheath and strength of fixation of different sheathed core bones in BASHTI technique. The comparison will be performed in controlled groups. The study hypothesized that sheathed core bone could increase the strength of the fixation during the insertion process and make this technique more feasible.

## Methods

### Preparation of samples and test material

**Figure 1.**
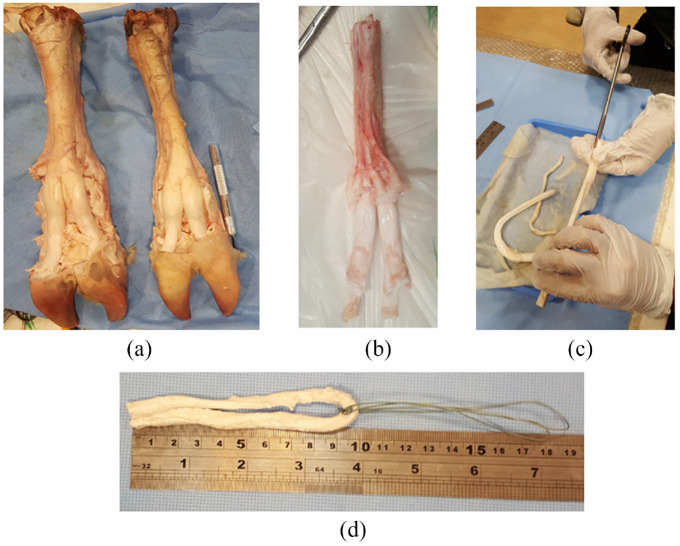
Harvesting procedure of tendon from bovine hoof samples. Fresh bovine hooves (a); Harvested digital tendon(b); Trimming a tendon (c); Looped tendon graft (d).

Studies showed that the mechanical properties of bovine digital tendon are suitable enough to be replaced with the human hamstring tendon when an ACL simulation is carried out in laboratory conditions.^10,20^ The current study used fresh bovine hooves from the same race and age to carry on an experimental examination. The tendon samples were harvested considering the local ethical policy and approved standard for biological testing. Sample preparation and laboratory instruments were used for all the samples at the same lab conditions. Thirty-four digital flexor and extensor tendons were harvested from provided bovine hooves. All tendons were harvested in the same way, sized to 7.5 mm diameter, using gage template, and stored at −20°C for not more than a few days to setup the actual experiment. The test was performed at room temperature (28°C) and 24% humidity. The tendon was cut at length of 180–250 mm to create a looped tendon graft with a length of 90–125 mm.^10^ The harvesting procedure and preparation of graft tendons are shown in Figure 1.

To conduct this controlled study, Polyurethane foam blocks from Sawbones company (Pacific Research Laboratories, Malmo, Sweden) with a density of 320 kg/m^3^ were used as they have similar properties to young human cancelous bone.^15,16,21^

**Figure 2.**
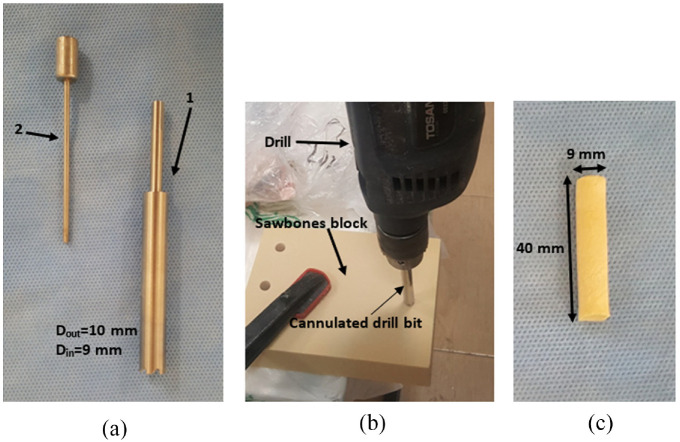
Manufactured cannulated drill bit to extract core bone plug (a), this drill bit consists of two parts: The main body(1) is used for drilling, and the pusher (2) is used to extract the bone plug out of the main body; Cutting a tunnel in the Sawbones block (b); Extracted core bone plug (c).

In the next step, a proper custom-made cannulated drill bit (Figure 2(a)) was designed for the test in order to create a tunnel in the Sawbones blocks (Figure 2(b)) and harvest the core bone plug that was used for graft fixation (Figure 2(c)).

The diameter of the cannulated drill bit was 9–10 mm (9 mm inside, 10 mm outside). Therefore, the bone plug diameter was 9 mm and tunnel diameter was 10 mm. The bone plug/tunnel length was 40 mm.^15^

**Figure 3.**
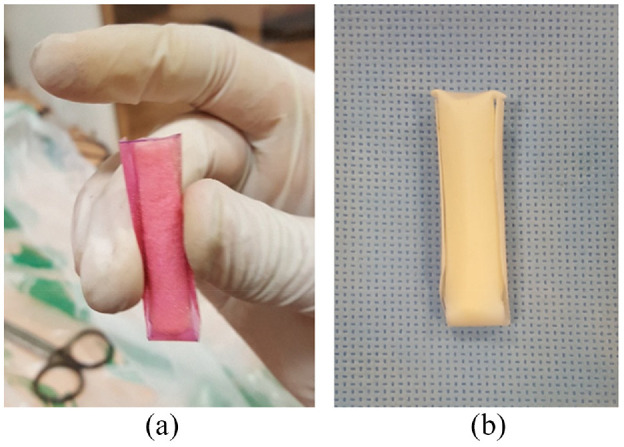
Bone plug sheathed with PVC (a) and bone plug sheathed with PTFE (b).

To arm the bone plug with a sheath, different sheaths were designed and prototyped. The sheaths were made of different polymer types. To find the appropriate candidate, mechanical parameters such as tensile strength, elongation, biocompatibility, flexibility, friction coefficient, resistance to impact, corrosion, cost, and density were evaluated.^22–27^ After assessment of these parameters, Polytetrafluoroethylene (PTFE) and Polyvinyl Chloride (PVC) polymers were selected for the sheath material.^22–27^ In the next step, the geometry of the sheath was designed based on what Saithna et al.^28^ suggested. Sheaths with 0.1 and 0.2 mm thickness were considered for final design. After designing, the samples were produced, and the bone plugs were sheathed, as shown in Figure 3. For ease of the bone plug insertion into the tunnel, the edge of the bone plug was slightly rounded.

**Figure 4.**
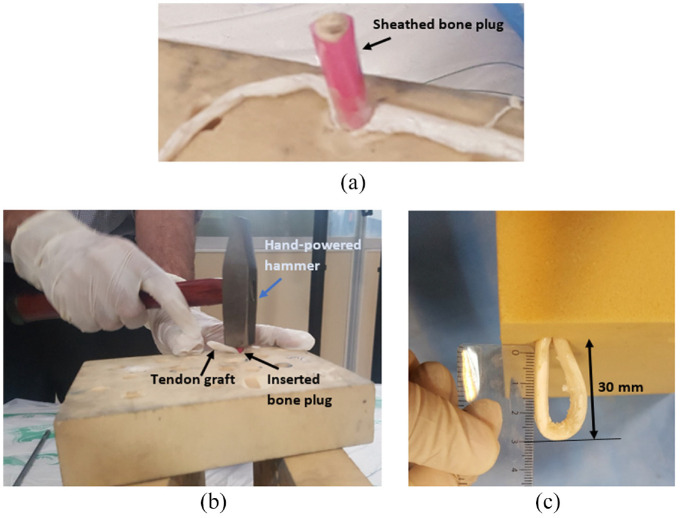
Placing sheathed bone plug between the looped tendon (a); Using a hand-powered hammer to insert the bone plug into the tunnel (b); The gage length of the looped tendon, similar to the length of an intact ACL (c).

To establish a BASHTI fixation, a looped tendon passed through the tunnel created in Sawbones blocks by using a surgical thread as a guide. The sheathed bone plug was then placed between the looped tendon (Figure 4(a)) and inserted into the tunnel using a hand-powered hammer (Figure 4(b)). It is noteworthy that in the insertion process, the gage length of the looped tendon was kept at 30 mm^13^ (Figure 4(c)). This is similar to the length of an intact ACL.^29^

### Test procedure

Thirty-four samples were tested: 10 for PVC and 24 for PTFE samples. Ten samples were tested using 0.1 mm thick PVC and PTFE groups. Due to clinical advantages, we observed for PTFE sheaths during the tests, we continued the 0.2 mm thickness sheaths only for PTFE group. In experiments involved with the 0.2 mm thick PTFE group, some sheaths were jammed into the tunnel during the insertion process and did not allow the bone plug to be fully inserted (labeled as the incomplete group). Therefore, 0.2 mm thick PTFE group were divided into two subgroups (i.e. complete and incomplete groups) to have a better understanding of the groups in which have the same mechanical properties. Fourteen tests were carried out for 0.2 mm PTFE group to get reliable results.

After fixing the tendon graft into the tunnel, a servo-hydraulic testing machine (Amsler HCT 25-400; Zwick/Roell AG, Germany) was used to evaluate the fixation strength of the samples. The machine was able to apply simple and cyclic tensile forces. This machine is designed for loads up to 25 kN. Depending on the design of the overall testing system, test frequencies of well over 100 Hz can be achieved. Also, 10 kHz control frequency for precise control, enables rapid reaction to spontaneous events.

**Figure 5.**
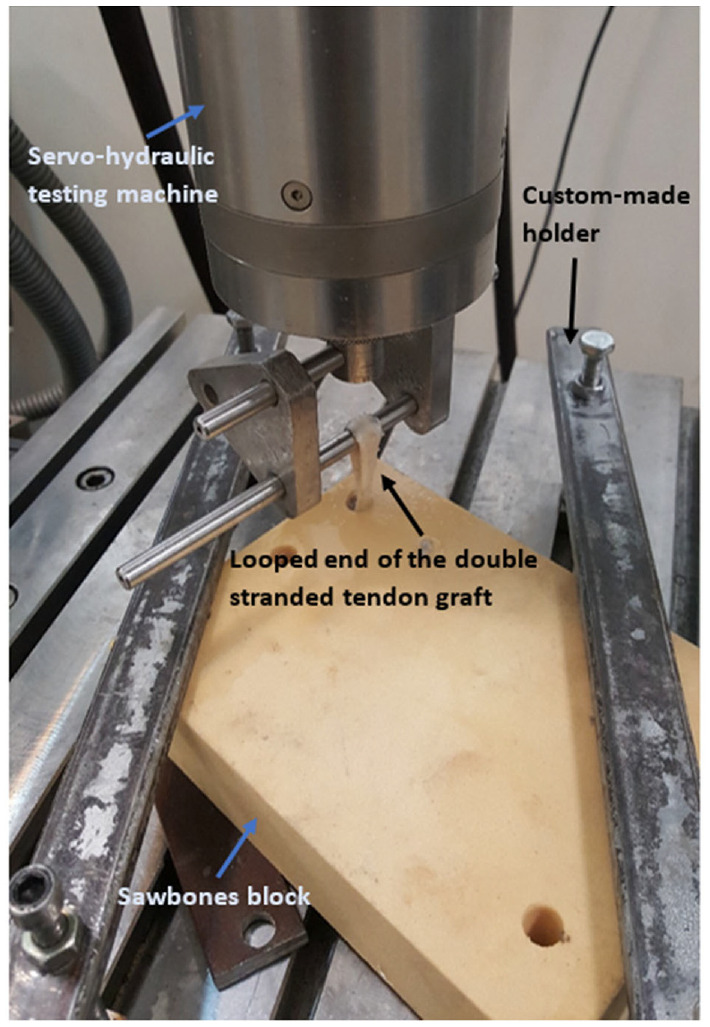
A sample mounted on the mechanical testing machine under cyclical test.

The loading process was applied in three different steps. In the first step, a preload was applied. This preconditioning load makes collagen fibers straight in the direction of applied forces.^30^ This load was carried out at 1 Hz frequency with a range of 5–20 N for 10 cycles. In the second loading step, the sample was under the main cyclic load, simulating forces applied to the ACL during normal walking activity. The load was carried out at 1 Hz frequency with a range of 40–120 N for 100 cycles.^15,16,31^ After completion of cyclic loading, the third loading step was applied. At this stage, samples are placed under a simple tensile loading. The tensile rate was 20 mm/s and continued until failure (Figure 5). The direction of the applied load was parallel to the tunnel axis. This condition leads to maximum pull-out force (axial loading), which is a worst-case scenario for an ACL which is under loading.^32^ During the testing process, saline solution was sprayed on the sample to keep the specimen moist and maintain the mechanical properties of the specimens.

### Outcome measures

According to previous studies,^15,33^ fixation rigidity is equal to the displacement difference between the 1st and 100th oscillations in the main cyclic loading. Also, the displacements at linear (elastic) region and non-linear (plastic) region of simple tensile loading is related to the third loading step. If the rigidity of the structure exceeded 10 mm, it could be said that the structure had lost its functionality, or the fixation failed.^33^ The 10 mm criterion is our understanding of the failure threshold after many experimental examinations performed by our team on ACL reconstruction using BASHTI method and if it exceeds that limit, we consider the graft as a failure and we don’t measure the pull-out force as in this case the tendon graft has already lost its functionality.

Yield force is considered as the end point of the linear region at the force-displacement curve. The slope of the linear region, which was related to elastic deformation, was also considered as the value of fixation stiffness. Moreover, ultimate fixation strength (ultimate force), were considered as the peak point of the force-displacement curve. Further to these considerations, the displacement at yield point up to the ultimate force (displacement at non-linear region) should be less than 5 mm. This displacement could be considered as fixation displacement.^33,34^ Therefore, if this displacement exceeds the value, the constructed graft might lose its function and it may result in an excessive laxity in the body.

### Statistical analysis methods

When the value of a scaling component in the test statistic is known, a *t*-test is typically used. When the scaling term is unknown and is substituted with an estimate based on the data, the test statistics follow a Student’s *t* distribution. So, the 95% confidence interval of the results were calculated using Student’s *t* distribution. Also, probability value (*p*-value) was considered to determine whether the differences between results are significant or not. If the *p*-value was equal or less than 0.05, it means the differences between two groups were significant, with 95% confidence.

### Ethical approval

All procedures performed in studies involving animal tissues were in accordance with the ethical standards of the Sharif Ethics Committee and with the 1964 Helsinki declaration and its later amendments or comparable ethical standards.

## Results

**Figure 6.**
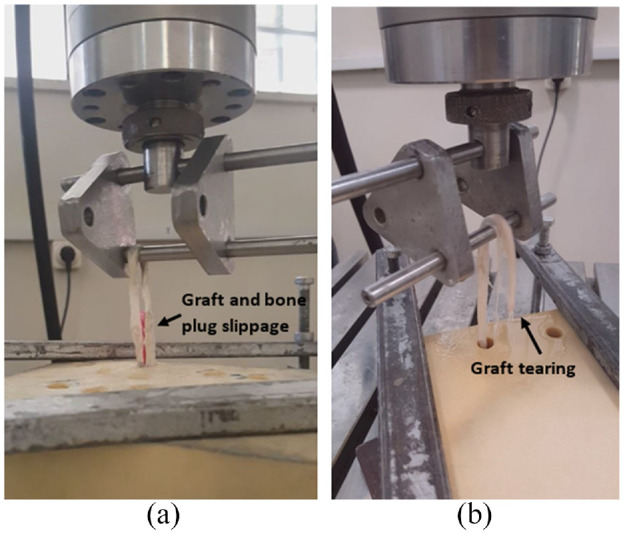
Failure modes in the experiments. Slippage in tendon graft in a PVC sheathed specimen (a); Rupture in tendon graft in a PTFE sheathed specimen (b).

The experimental results showed two types of failure modes in tests. The first failure mode was related to the slippage of the graft/core bone plug at the fixation site. The second mode was related to the tearing of the tendon under tensile loading. Figure 6 shows the two failure modes observed in this study. The sheath with 0.1 mm PTFE sheath had better fixation and mechanical strength conditions than 0.1 mm PVC sheath, and it was also more feasible in the assembly and insertion process. As a result, PTFE sheath was chosen as a feasible design^22–27^ and continued a further examination on it. Besides, the PTFE sheath with 0.2 mm thickness was also tested in order to validate the sheath thickness.

The failure mode in different groups is summarized in Table 1. In this regard, most of the samples of the PVC sheath group failed due to slippage failure mode. While in the PTFE sheath group, tearing was the dominant failure mode.

**Table 1.** Failure mode in different groups.

Groups	Sheath design	Failure mode	
		Slippage(number)	Tearing(number)
1	0.1 mm PVC	7	3
2	0.1 mm PTFE	4	6
3	Complete 0.2 mmPTFE	2	6
3	Incomplete 0.2 mmPTFE	3	3

Table 2 shows the rigidity and displacement in different conditions.

**Table 2.** Displacement and rigidity of fixation groups. Rigidity of fixation is equal to the displacement difference between the 1st and 100th oscillations in the main cyclic loading.

Groups	Displacement at the1st cycle (mm)	Displacement at the100th cycle (mm)	Rigidity offixation (mm)	Displacement at linear region of simpletensile test (mm)	Displacement at non-linearregion of simple tensile test (mm)
1	2 ± 0.49	6.31 ± 3.08	4.31 ± 2.8	1.57 ± 1.23	4.94 ± 3.79
2	1.55 ± 0.4	4.17 ± 3.01	2.62 ± 2.65	2.31 ± 1.4	3.30 ± 2.53
3 (overall)	3.5 ± 4.14	6.67 ± 5.64	3.17 ± 2.44	2.37 ± 1.25	2.53 ± 2.82

**Figure 7.**
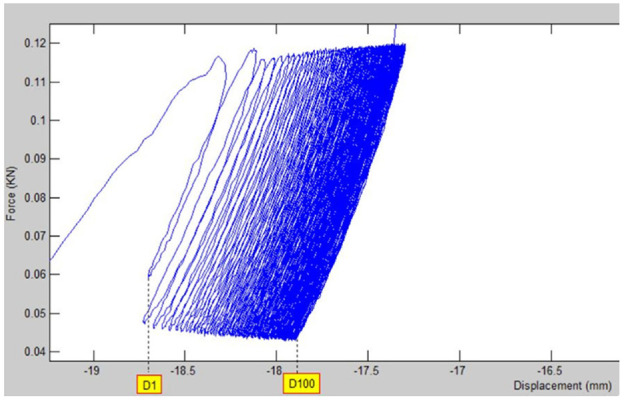
Graph of cyclic loading test.

A typical output of main cyclic loading step is shown in Figure 7.

The area under the curve indicates lost energy due to the internal friction of the material, and it is noted that in cyclic tests the hysteresis loops gradually become slimmer, and after a specific number of loops the curve stops moving forward, and the lost energy reaches to its minimum, and it shows that we have reached to stable condition and there would be no more creep in samples.^30^

Table 3 shows the mechanical properties of fixation for different groups.

**Table 3.** Mechanical properties of fixation groups. Yield force is considered as the end point of the linear region at the force-displacement curve. Ultimate force was considered as the peak point of the force-displacement curve. The slope of the linear region was also considered as the stiffness.

Groups	Sheath design	Number of samples	Yield force* (N)	Ultimate force* (N)	Stiffness* (N/mm)
1	0.1 mm PVC	10	186.37 ± 36.93	235.95 ± 56.9	93.36 ± 20.68
2	0.1 mm PTFE	10	304.47 ± 52.35	343.86 ± 94.2	114.62 ± 20.71
3	0.2 mm PTFE	14	276.64 ± 81.6	340.48 ± 109.03	99.95 ± 36.32
3	Incomplete fixation	6	206.62 ± 34.4	221.6 ± 36.52	66.99 ± 21.19
3	Complete fixation	8	323.31 ± 69.23	420.02 ± 46.64	126.16 ± 23.88
Dehestaniet al.’s^15^ study	No sheath	10	188. 7 ± 82.37	363 ± 145.83	93.9 ± 26.13

* Force and stiffness values are presented as their mean values with standard deviation (SD).

Statistical analyses were carried out to assess the results of the test groups. The probability values for each of the mechanical parameters were compared for two groups and were presented in Table 4. If this value was less than 0.05, it meant there was a significant difference (with 95% confidence) between two groups.

**Table 4.** Probability value of mechanical properties for different groups.

Compared groups	*p*-Value for yield force	*p*-Value for ultimate force	*p*-Value for stiffness	*p*-Value for rigidity
1 & 2	0.0001	0.0062	0.0339	0.1826
2 & 3 (Incomplete fixation)	0.0012	0.0091	0.0010	0.0578
2 & 3 (Complete fixation)	0.5197	0.0537	0.2884	0.4586
1 & 3 (Incomplete fixation)	0.2943	0.5805	0.0478	0.4725
1 & 3 (Complete fixation)	0.0001	0.0001	0.0066	0.0368
3 (Incomplete fixation) & 3 (Complete fixation)	0.0027	0.0001	0.0007	0.0040

## Discussion

Dehestani et al.^15,16^ investigated the effect of bone density in BASHTI method and found that the polyurethane foam blocks with a density of 320 kg/m^3^ can be used to simulate human bone. They confirmed that this density could represent young people with relatively high bone density. Therefore, Sawbones blocks with the same density (320 kg/m^3^) were used in this study to represent individuals with highly dense bones (young group). In addition, by using these sawbones blocks, we will have a controlled study without variations in mechanical properties of different animal bones because of anatomical, and physiological differences.

It was found that by using a sheath during the insertion process in BASHTI technique, due to a lower friction at the contact zone between the core bone and the tunnel wall, a lower number of hammer impacts were needed to insert the core bone into the tunnel. Therefore, the insertion process became easier and the damage to the core bone reduced significantly.

**Figure 8.**
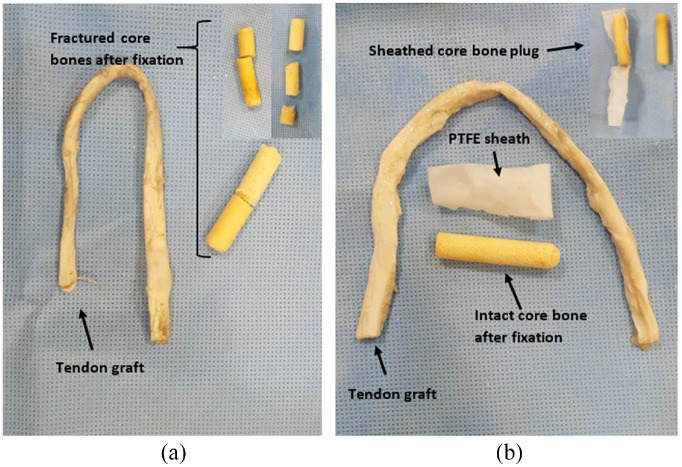
Tendon and core bone plug samples after fixation and mechanical testing without using sheath (a); Tendon and sheathed core bone plug samples after fixation and mechanical testing (b).

In other words, during the fixation of the bone plug without a sheath, because of the high friction between the bone plug and the tunnel, the plug could not easily be inserted into the tunnel, and a massive impact should be applied on the bone plug. This impact caused failure and fracture on the bone plug^15^ and could result in improper initial fixation. Typical fractured samples during the insertion process and tendon graft after the mechanical testing are shown in Figure 8(a).

Moreover, during the insertion phase, a massive impact on the bone plug was needed. This could result in an extensive pressure on the tunnel wall, which could lead to a tunnel widening. Also, the bone plug edge could damage the graft fibers during the insertion process.

The results from those samples with a sheathed bone plug show that the bone plug was safely survived following the fixation, as shown in Figure 8(b).

When a sheathed bone plug was used, due to lower friction at the insertion step, the bone plug entered the tunnel easier, and therefore, a lower impact force was required for its insertion. This provided a fixation with higher quality. Moreover, the risk of fracture was lower in the bone plug, which meant that the entire bone plug could enter into the tunnel during the insertion phase. The results showed that, by full insertion of the bone plug into the tunnel, the contact surface between the bone plug and the tunnel wall reached to its maximum level. As a result, the fixation strength increased significantly (Table 3).

According to the statistical analyses presented in Table 4, the differences between the mechanical properties of the second group (0.1 mm PTFE) and the third group (0.2 mm PTFE) with complete fixation were insignificant (*p* > 0.05). In similar conditions, the mechanical properties of the first group (0.1 mm PVC) and the third group with incomplete fixation were close too. The main factors that affect these results are mechanical properties of the sheath (i.e. differences between PTFE and PVC polymer) and the level of bone plug insertion into the tunnel (i.e. core bone engaged length).

Moreover, the mechanical properties of the second group and third group with complete fixation were remarkably higher than the first group and the third group with incomplete fixation and the differences between them were significant (*p* ≤ 0.05). Regarding the first group, although minimizing the friction facilitates the insertion process, the sheath properties in the first group (PVC polymer) reduced the friction force more than needed and caused the fixation unstable and led the fixation to failure at a low pull-out force. This is because of the lower friction generated by the PVC polymer rather than PTFE polymer, resulted a slippage at early stage of pull-out loading, and made the reconstruction challenging for this polymer. As a result, we excluded PVC for 0.2 mm thickness and only analyzed the 0.2 mm PTFE sheath for further analyses.

The diameter of tunnel after drilling was measured and it matched our anticipated diameter (10 mm) and heating and other factors had minimal effects on the results. On the other hand, after fixation of bone plugs into the tunnel and mechanical testing, some minor enlargements were seen in the tunnel diameter due to using hammer and pull-out testing; but the differences with the intact tunnel were insignificant. In addition, we divided the 0.2 mm group into two different groups since the jamming parameter played a vital role in the mechanical properties’ outcomes, and to better interpret the data we had to divide this group into two different groups.

In the third group with incomplete fixation, the high impact force that was applied to the bone plug during the insertion process, caused a damage to the tendon fibers and the tunnel wall. Consequently, the compressive force which was needed to push the graft into the tunnel wall decreased and reduced the structure fixation strength. Additionally, since the whole core bone was not inserted into the tunnel (some parts of it left outside or destroyed due to the high impacts of the hammer) the core bone engaged length decreased. So, it decreased the compressive force (friction force) to push the graft into the tunnel. The authors anticipate that by using auto hammer with specified frequencies, we could avoid this problem resulting in incomplete fixation.

Therefore, to have the best fixation strength, looking for an optimum condition that the fixation failed neither due to the weakness of the fixation and slippage of the graft nor due to the excessively compressing forces to cut the tendon fibers was suggested. As a result, it was found that among all groups, the second group (0.1 mm PTFE) and the third group (0.2 mm PTFE) with complete fixation were suitable for this technique in terms of yield force, ultimate force, stiffness, and rigidity. Although, the mean value of the mechanical properties in the third group with complete fixation was higher than the second group, the values were not significantly different (*p* = 0.5197, 0.0537, 0.2884, 0.4586 for yield force, ultimate force, stiffness, and rigidity, respectively). The main advantage of the third group was its higher fixation strength, and the advantage of the second group was complete insertion of the core bone into the tunnel in all specimens.

It is noteworthy to mention that the yield force values of the second group (0.1 mm PTFE) and the third group (0.2 mm PTFE) with complete fixation were significantly higher than the previous study^15^ in which no sheaths were used (*p* = 0.0015 and 0.0020 for the second group and third group with complete fixation, respectively). In addition, the average stiffness value for the second group was higher than that of no sheath’s study,^15^ but the differences were not considerable (*p* = 0.065). Also, the stiffness value of the third group with complete fixation was significantly higher than that of no sheath’s study^15^ (*p* = 0.0157) with 95% of confidence. Therefore, it could be concluded that using PTFE sheath improves the mechanical properties of the fixation in BASHTI technique and this is in line with the study’s hypothesis.

In this technique, we used PVC and PTFE polymers to see the effects of using a sheath on the mechanical properties at the fixation site. Now that, we realized using sheaths would be beneficial for our technique, we could use biodegradable materials with similar mechanical properties to our polymers (e.g. PEEK, PLGA) which are easy to produce and apply on sheaths due to their prevalent clinical usages in healthcare.

The authors believe the sheathed technique could be upgraded to reach a fixation strength above the interference screws fixation technique. Finally, it should be noted that, BASHTI technique advantages, such as higher healing speed and lower cost, could make this technique an attractive choice for the ACL reconstruction.

## Limitations

Following limitations have been involved with current study. It is recommended to consider those in future studies.

- The use of in vivo animal model and evaluation of healing process.
- Study the effect of biodegradable sheaths on the fixation.
- The use of an auto hammer with adjustable impulse to insert the core bone.

## Conclusions

This study showed that using sheathed bone plug facilitates the fixation process in BASHTI technique and enhances the mechanical properties of the fixation and makes this technique more feasible for ACL reconstruction. It was also found that the 0.1 mm PTFE sheath and 0.2 mm PTFE sheath with complete fixation provides better fixation strength than 0.1 mm PVC sheath. The outcomes showed that the inserted length of the bone plug inside the tunnel (core bone engaged length) has a direct effect on the fixation strength.
